# 476. A Global Survey of Countermeasures Against the COVID-19 Pandemic Among Solid Organ Transplant Centers

**DOI:** 10.1093/ofid/ofab466.675

**Published:** 2021-12-04

**Authors:** Aasith Villavicencio, Mohammed Raja, Deepali Kumar, Deepali Kumar, Lara Danziger-Isakov, Lara Danziger-Isakov, Nicole Theodoropoulos, Michele I Morris, Giselle Guerra, Lilian M Abbo, Yoichiro Natori

**Affiliations:** 1 University of Miami Miller School of Medicine, Miami, Florida; 2 University of Miami Miller School of Medicine/Sylvester Comprehensive Cancer Center, Miami, Florida; 3 University Of Toronto, Toronto, ON, Canada; 4 Cincinnati Children’s Hospital Medical Center, Cincinnati, OH; 5 University of Massachusetts, Worcester, Massachusetts; 6 University of Miami / Jackson Memorial Hospital, Miami, Florida; 7 University of Miami Miller School of Medicine & Jackson Health System, Miami, Florida; 8 Jackson Memorial Hospital/Miami Transplant Institute, University of Miami Miller School of Medicine, Miami, FL

## Abstract

**Background:**

Solid organ transplantation (SOT) profoundly impacts vulnerable recipients with chronic end organ diseases. The COVID-19 pandemic disrupted healthcare systems, including organ transplants. We aimed to evaluate the responses of SOT centers to COVID-19 at the beginning of the pandemic around the world.

**Methods:**

We conducted a web-based survey amongst transplant centers, sent to members of The American Society of Transplantation Infectious Diseases Community of Practice Group, between April and May 2020. The survey included basic information of each transplant center (number and types of transplants in 2019), the countermeasures employed against COVID-19 such as timing of postponing of transplantation, and management of outpatient clinics including implementation of telemedicine and screening for in-person visits.

**Results:**

A total of 65 centers from 19 countries responded (Table 1). Regarding the percentage of hospitalized patients with COVID-19 at the time of the survey, 39 (60%) centers reported < 10%, two centers reported > 80%. All centers reduced their services to some extent as shown in Table 2. Centers reported postponing living donor kidney transplant (50/58, 86%), deceased donor kidney transplant (20/57, 35%), living donor liver transplant (32/42, 80%), deceased donor liver transplant (17/41, 41%), lung transplant (20/31, 65%), heart transplant for LVAD (18/33, 55%) and non-LVAD patients (18/33, 55%). In March and April 2020, cancellation of pre- and post- transplant clinics were reported by 36/64 (56%) and 17/65 (26%) centers. Postponing clinic appointments were reported by 56/65 (86%) centers. Most institutions (54/64, 85%) used telemedicine. Screening for COVID-19 for clinic visits was done by telephone, in-person questionnaires and/or temperature checks.

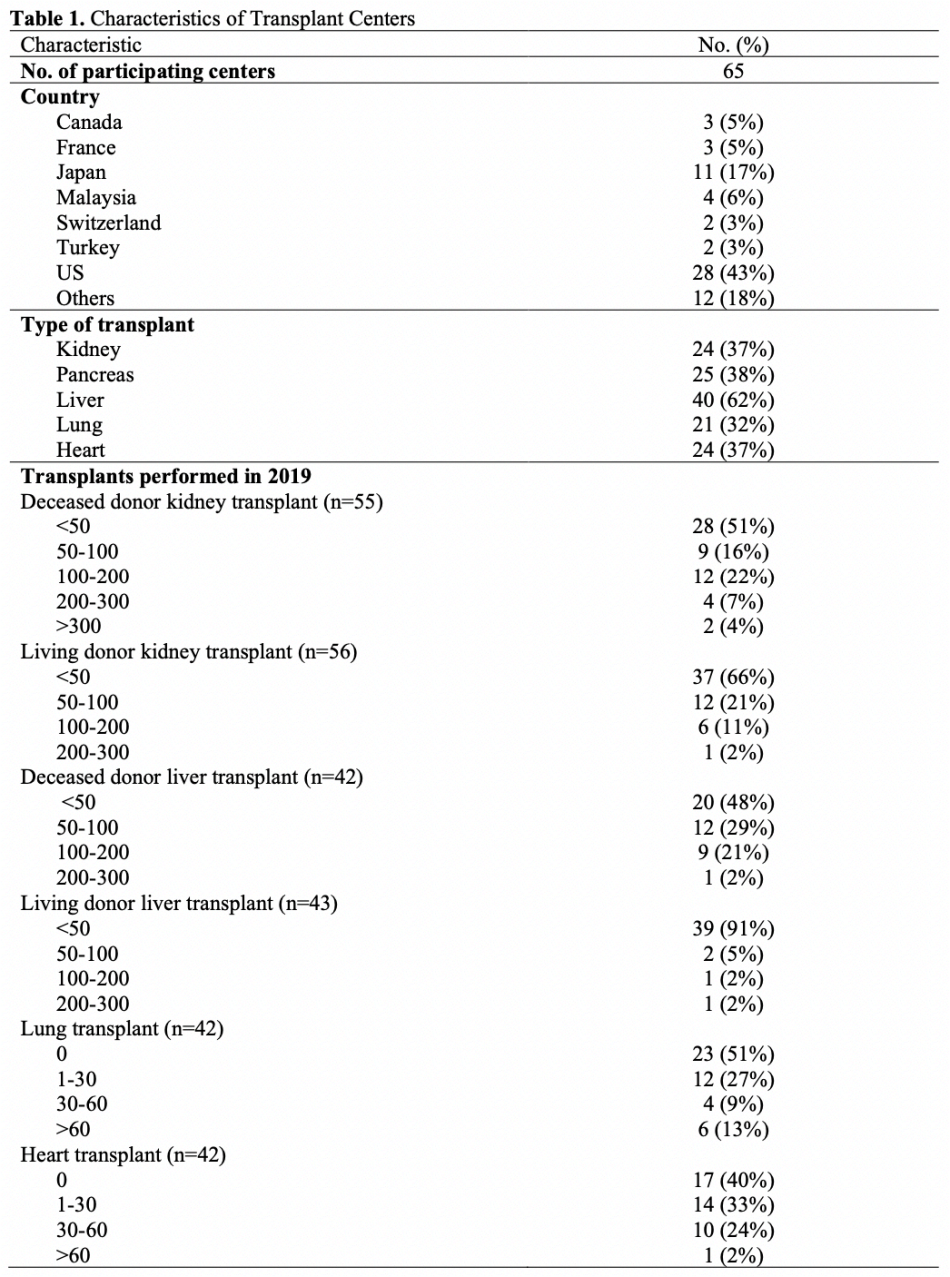

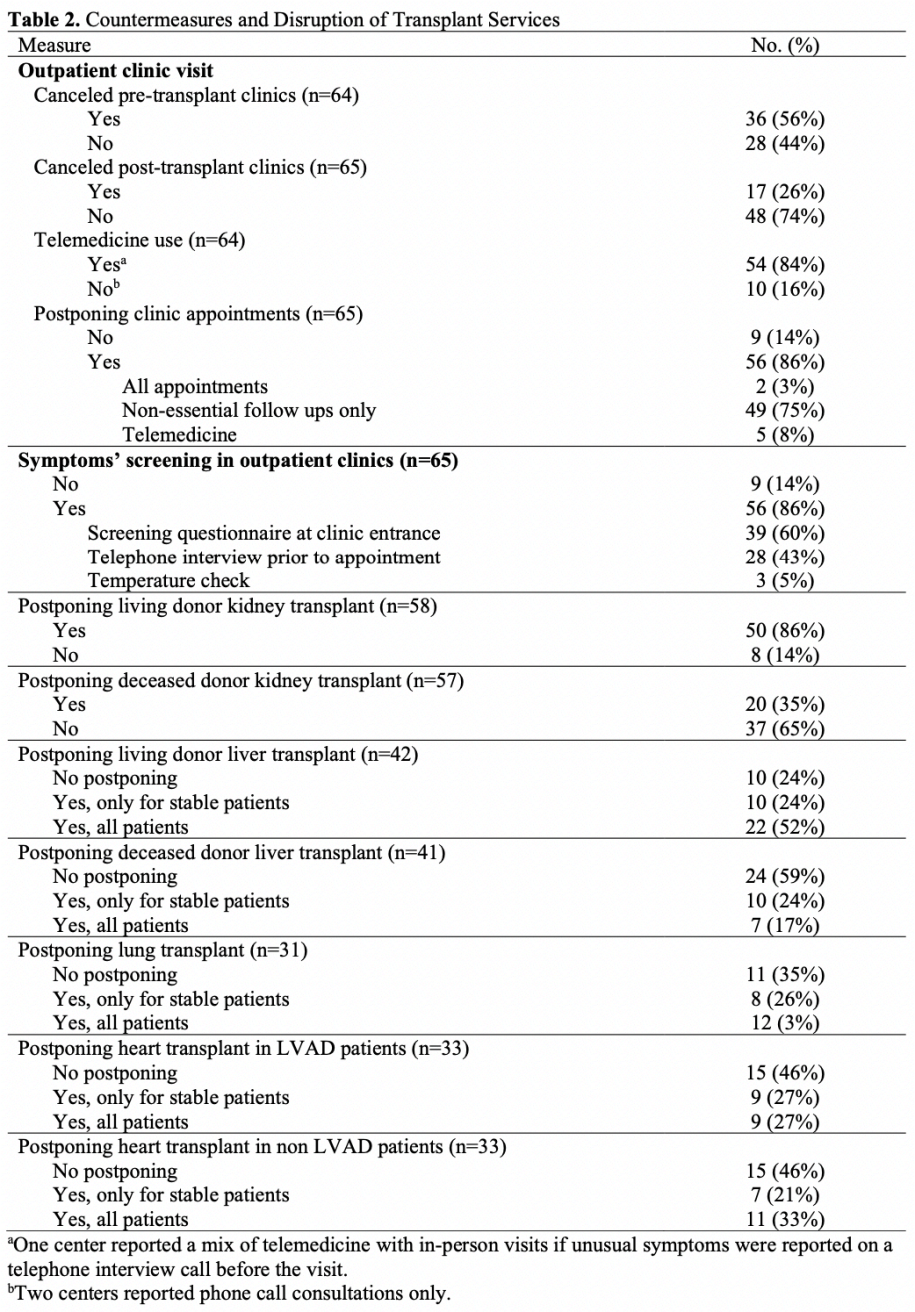

**Conclusion:**

During the early phase of the pandemic, when management strategies were highly uncertain, non-urgent and living donor transplants were frequently postponed. Emergent liver transplants continued regardless. These findings could help us navigate SOT in future epidemics. Limitations included a small sample and lack of assessment of clinical outcomes from postponing SOT.

**Disclosures:**

**Deepali Kumar, MD, MSc, FRCPC**, Astellas (Individual(s) Involved: Self): Speakers’ bureau; Atara Biotherapeutics (Individual(s) Involved: Self): Grant/Research Support; GSK (Individual(s) Involved: Self): Consultant, Grant/Research Support; Merck (Individual(s) Involved: Both Myself and my Spouse/Partner): Advisor or Review Panel member, Grant/Research Support; Oxford immunotec (Individual(s) Involved: Self): Consultant, Grant/Research Support; Pfizer (Individual(s) Involved: Self): Speakers’ bureau; Roche (Individual(s) Involved: Self): Consultant, Grant/Research Support; Sanofi (Individual(s) Involved: Self): Advisor or Review Panel member; Shire/Takeda (Individual(s) Involved: Both Myself and my Spouse/Partner): Advisor or Review Panel member, Grant/Research Support **Lara Danziger-Isakov, MD, MPH**, Ansun (Individual(s) Involved: Self): Scientific Research Study Investigator; Astellas (Individual(s) Involved: Self): Scientific Research Study Investigator; Merck (Individual(s) Involved: Self): Consultant, Scientific Research Study Investigator; Pfizer (Individual(s) Involved: Self): Scientific Research Study Investigator; Shire (Individual(s) Involved: Self): Consultant, Scientific Research Study Investigator; Viracor: Grant/Research Support

